# Nano-polymeric curing agents for enhancing water stability in sandy soils: A sustainable approach for ecological slope protection

**DOI:** 10.1371/journal.pone.0330112

**Published:** 2025-08-14

**Authors:** Shanshan Zhao, Aijun Chen, Xiong Shi, Zheng Li

**Affiliations:** 1 Guangzhou Railway Polytechnic, 198 Kede Avenue, Guangzhou, Guangdong 511300, China,; 2 School of Architecture and Transportation Engineering, Guilin University of Electronic Technology, Guilin, China; China University of Mining and Technology, CHINA

## Abstract

The susceptibility of sandy soil slopes to erosion, particularly during rainfall events, poses significant challenges for soil conservation and ecological slope protection. This study explores the potential of nano-polymeric curing agents (NPCA) as a sustainable solution to enhance water stability and slope integrity. Reinforcement depth experiments were conducted to determine the optimal application depth of NPCA, while permeability and erosion tests assessed its impact on water retention and soil stability. Advanced analytical techniques, including scanning electron microscopy (SEM) and Fourier-transform infrared spectroscopy (FTIR), were employed to examine the interactions between NPCA and soil particles. Results show that a 3% NPCA content (mass ratio) achieves the maximum reinforcement depth of 23 mm. Within the optimal reinforcement range (mass ratio < 3%, concentration < 17%), increasing NPCA content enhances soil permeability, reduces the disintegration coefficient, and improves erosion resistance. NPCA encapsulates soil particles, filling pore spaces and binding them through van der Waals forces and hydrogen bonds, forming a durable, elastic membrane that enhances surface stability and water resistance. These findings suggest that NPCA treatment creates a stable, permeable, and breathable environment, crucial for promoting vegetation growth on sandy slopes and offering an effective, sustainable approach to ecological slope protection.

## 1. Introduction

Sandy soil slopes, characterized by loose topsoil and poor water-holding capacity, are highly susceptible to rain-induced erosion. This erosion often leads to slope destabilization, presenting significant management challenges. Traditional slope protection techniques, such as shotcrete, are increasingly inadequate for ecological conservation needs [1,2], resulting in a growing emphasis on ecological slope protection methods, such as vegetation [3–5]. Vegetative methods are effective because plant stems [6] and roots [7–9] can intercept runoff and reinforce soil, respectively. However, these slopes are vulnerable to rainfall during the early construction stages, often leading to vegetation failure.

Recent research has highlighted polymer curing agents as promising soil reinforcement materials, offering improvements in both geotechnical properties and ecological compatibility [10–13]. Studies have demonstrated the effectiveness of these polymers in enhancing sandy soil's mechanical properties and resistance to water-related forces [14,15]. Notably, SH sand fixer [16–18] and synthesized high-molecular-weight polyacrylamide [19] have achieved significant improvements in soil resistance.

In the past five years, the development of biopolymer-based and nano-enhanced polymer stabilizers has attracted increasing attention due to their environmentally friendly nature and functional versatility. For instance, Al-Mahbashi and Almajed [20] demonstrated that biopolymers like xanthan gum significantly improved the water retention and aggregate stability of sandy soils, making them more conducive to plant growth in arid environments. Similarly, Feizi et al. [21] investigated a polymeric nanocomposite for sand stabilization and reported enhanced erosion resistance and mechanical strength, particularly under wind and rainfall simulations.

Boaventura et al. [22] applied an eco-friendly acrylic polymer and found that even at low dosages (1–3%), the polymer could form a durable crust on the sand surface, reducing soil loss by over 90% in simulated rainfall tests. Lu et al. [23] used polymer amendments in seasonally frozen slopes and reported that these materials not only improved frost resistance but also acted as a stable substrate for vegetation growth. In another study, Wang et al. [24] optimized the application of chitosan under different acid concentrations, showing that the long-term bonding strength could be effectively tuned without compromising biodegradability.

Although these studies [25–30] have effectively enhanced selected properties such as strength, erosion resistance, and moisture retention, soil stability is a multi-parameter phenomenon. It relies on the synergistic coordination of various factors including particle bonding, pore structure regulation, and ecological compatibility. Moreover, most existing research still focuses on the engineering performance of polymer-stabilized soils, with limited exploration of how these amendments contribute to building a harmonious and resilient ecological substrate, especially in the early stages of vegetative slope restoration. The current study aims to bridge this gap by proposing a nano-organic polymer (NPCA) that not only reinforces sandy soil but also supports long-term ecological functions.

This study introduces the nano-organic polymer NPCA as a novel reinforcement material for sandy soil slopes. NPCA appears as a white emulsion at room temperature, with a density of 1.01 g/cm^3^. Its pH ranges between 6 and 7, indicating weak acidity. The solid content is 41%, and the viscosity ranges from 80,000–100,000 mPa·s. The peak molecular weight is approximately 625,531 Daltons, suggesting that NPCA with a higher molecular weight exhibits greater viscosity.

We examine NPCA’s reinforcement depth and diffusion in sandy soil, its impact on soil permeability, and its resistance to water erosion through various tests, including infiltration, disintegration, and simulated rainfall scouring. Using scanning electron microscopy and infrared spectroscopy, we explore NPCA’s interaction with sandy soil and its role in enhancing water stability and ecological slope protection. Our findings provide valuable insights for ecological slope conservation, especially in regions like South China, and contribute to the development of new polymer-based soil protection technologies.

## 2. Materials and methods

### 2.1. Materials

The sandy soil used in the experiment was sourced from the Tianhe District in Guangzhou City. The sampling was conducted with prior authorization from the site management. Since the collection did not involve ecological resources or protected areas, no specific government permits were required.

**Fig 1:**
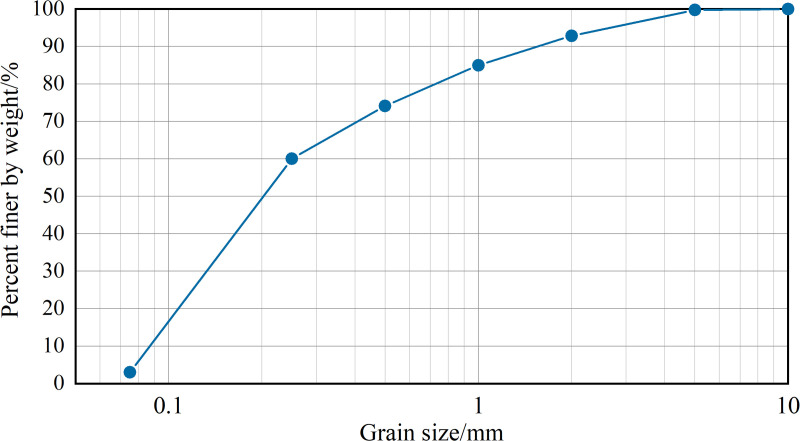
Particle size distribution curve of sandy soil for test.

The basic physical properties of the sandy soil were determined in accordance with the Standard for Geotechnical Test Methods (GB/T 50123−2019). The results are presented in Table 1 and Fig 1.

**Table 1:** Basic physical properties of sandy soil.

Property	Value
Type of soil	sand
Specific gravity	2.64
Median particle size D_50_/mm	0.202
Uniformity coefficient *Cu*	2.88
Curvature coefficient *Cc*	0.81
Maximum dry density/(g/cm^3^)	1.70
Minimum dry density/(g/cm^3^)	1.34
Maximum void ratio	0.97
Minimum void ratio	0.55

The nano-organic polymer NPCA used in the experiment is a water-resistant modified polyester with the molecular formula [CH2CHCOOCH3]n. The preparation process of NPCA is as follows [31]: mix at least one polyol containing one or more secondary or tertiary hydroxyl groups with at least one polyacid uniformly. Under the influence of a catalyst, a condensation reaction is carried out, and at least one polyacid is added in the later stages of the reaction.

The polymer chains contain hydroxyl (-OH) and carboxyl (-COOH) functional groups, facilitating connections with negatively charged soil particles. A molecular weight distribution value of 1.02 suggests that NPCA has a single-chain structure. The longer single-chain structure promotes contact between the active groups on the polymer chain and soil particles. Additionally, the bending and entanglement of the long-chain polymers further bind soil particles and improve the stability of the bond. NPCA is used for ecological slope reinforcement of the top layer of sandy soil, and its ecological properties can impact the soil's environmental condition. An Energy Dispersive X-Ray Fluorescence (EDXRF) analysis was conducted on NPCA, with results presented in Table 2.

**Table 2:** EDX analysis results of NPCA.

Element	C	O	Al	Fe	Cu	Pd	Au	(grand) total
Weight ratio/%	**43.62**	**47.62**	1.66	1.96	0.46	2.1	2.58	100

The Energy Dispersive X-Ray (EDX) analysis revealed that carbon (C), oxygen (O), and hydrogen (H) are the predominant elements in NPCA. Trace amounts of aluminum (Al), copper (Cu), and iron (Fe) were also detected, likely introduced as impurities during the synthesis of NPCA. Importantly, these findings suggest that NPCA’s application in reinforcing sandy soil is environmentally benign, as its degradation products are carbon dioxide (CO_2_) and water (H_2_O), posing no pollution risk.

### 2.2. Methods

The sandy soil collected from the site underwent a series of preparatory steps before testing. Initially, the soil was oven-dried at 105°C to remove natural moisture content. Once dried, the soil was gently milled to break down aggregates and passed through a 2 mm aperture sieve.

To simulate realistic field conditions while maintaining laboratory control, the target dry density of all test specimens was set at 1.6 g/cm^3^, and the moisture content was fixed at 15%. These values were selected based on standard Proctor compaction tests, which indicated that they fall within the optimal moisture content and maximum dry density range for the tested sandy soil. Therefore, these parameters represent an appropriate compaction state that balances workability and structural stability, while facilitating effective interaction between the polymer and soil particles.

To systematically investigate the influence of NPCA (nano-polymeric curing agent) dosage on the engineering properties of sandy soil, different dosage gradients were tailored for each test according to specific objectives. The grouping logic and corresponding NPCA concentrations for each experiment are summarized in Table 3.

**Table 3:** NPCA dosage schemes for different laboratory tests.

Test Type	NPCA Dosage (%)	Purpose
Reinforcement depth test	0.75, 1.5, 3.0, 6.0, 9.0	Broad range to identify optimal and threshold concentrations
Permeability test	0.75, 1.5, 3.0, 6.0, 9.0	Matched to reinforcement depth test for comparison
Disintegration test	0.5, 1.0, 2.0, 3.0, 5.0	Focus on lower range for sensitivity and resistance thresholds
Scrub test	0.5, 1.0, 1.5, 2.0	Evaluate erosion rate under practical dosage conditions

All samples used in the subsequent reinforcement depth test (2.2.1), permeability test (2.2.2), disintegration test (2.2.3), and erosion resistance test (2.2.4) were prepared using this standardized moisture content and dry density, thereby ensuring comparability and reliability of the experimental results.

#### 2.2.1. Reinforcement depth test.

For this test, 500g of sieved soil was accurately weighed and placed into six transparent sample tubes, each with a diameter of 100 mm and a height of 100 mm. The soil in each tube was compacted to a uniform height of 55 mm. Differing concentrations of NPCA water solution—0.75%, 1.5%, 3.0%, 6.0%, and 9.0% (mass ratio)—were prepared according to the designed water content of the sandy soil. These solutions were then evenly sprayed into the sample tubes. For control purposes, reference samples received an equivalent amount of water. All samples were left to condition at room temperature for three days.

The effectiveness of NPCA reinforcement was tested using the immersion method. This involved extracting the treated soil from each tube using a soil extraction ring cutter (Φ50.46 × 50 mm). The residual length of the soil specimen, post-immersion and stabilization, was measured to determine the effective depth of NPCA reinforcement.

#### 2.2.2. Permeability test.

This test aimed to evaluate the permeability of sandy soil treated with NPCA. Five different concentrations of NPCA aqueous solutions were prepared, corresponding to 0.75%, 1.5%, 3.0%, 6.0%, and 9.0% of the dry soil mass. The soil was sieved and then uniformly mixed with these solutions. For control (reference) samples, labeled CK, an equivalent quantity of water was used for mixing.

The specimens were prepared using the static pressure method, aiming for a dry density of 1.6g/cm^3^. Six sets of specimens were immediately formed, each set comprising three individual specimens to allow for parallel testing. Post-preparation, the specimens were placed in a constant temperature maintenance box set at 25°C for a period of two days.

Permeability testing followed the guidelines set forth in the Standard for Geotechnical Test Methods (GB/T50123-2019). Using a TST-55 penetrometer, a normal head test was conducted on the sandy soil specimens, each having an inner diameter of 61.8 mm and a height of 40 mm.

#### 2.2.3. Disintegration test.

For the disintegration test, 500g of sieved soil was measured and placed into six transparent sample tubes (100 mm diameter and 100 mm height). Each soil sample was compacted to a height of 55 mm. A series of NPCA aqueous solutions at concentrations of 0.5%, 1.0%, 2.0%, 3.0% and 5.0% were prepared. These solutions were sprayed over the compacted soil, maintaining a uniform application rate of 3L/m². Reference samples (CK) received no NPCA treatment. After spraying, all samples were conditioned at room temperature for three days.

**Fig 2:**
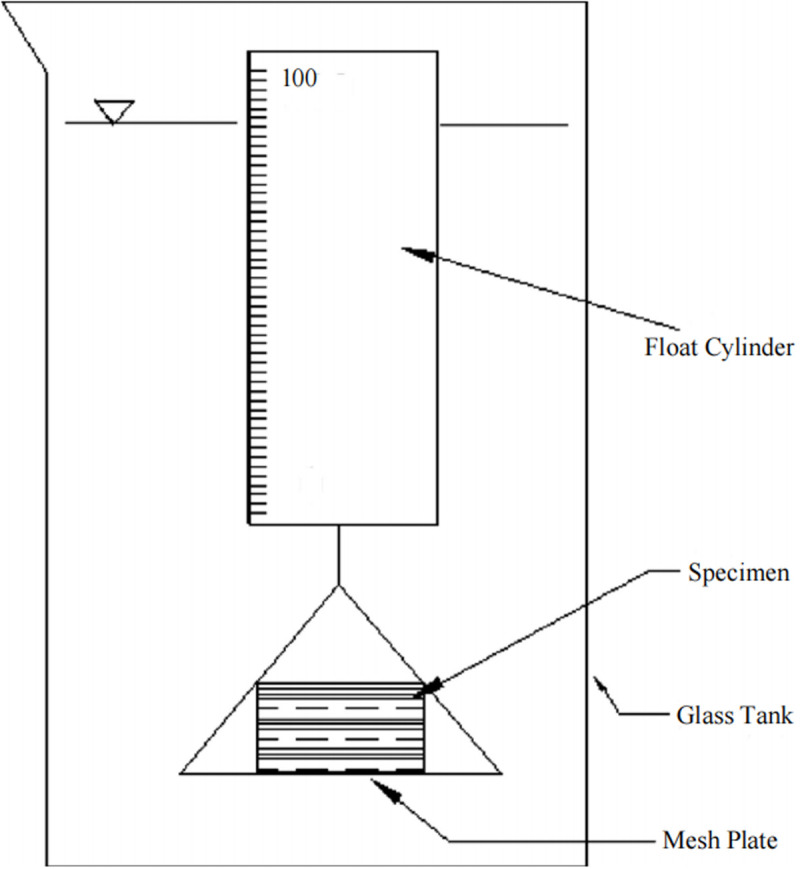
Jiang Dingsheng soil wetting instrument.

The disintegration resistance and soaking stability of the sandy soils were assessed using a modified Jiang Dingsheng soil wetting instrument. As shown in Fig 2, the apparatus consists of a glass cylinder filled with water, a precision balance (accurate to 0.05 g), a mesh plate (1 cm × 1 cm aperture), support rods, connecting wires, and hooks. Soil samples for testing were extracted from the tubes using a soil extraction ring knife (Φ50.46 × 50 mm).

The average disintegration rate, a measure of the sandy soil's resistance to disintegration, was calculated using the following formula:


$$v(t)=\frac{m_{0}-m_{t}}{t}$$ (1)

Where  v(t) is average disintegration rate (g/min); $m_{0}\;$is initial balance reading (g); $m_{t}\;$is final balance reading (g); and t is immersion time (min).

Additionally, the disintegration coefficient, indicative of the soaking stability, was determined by:


$$K(t)=1-\frac{m_{t}-m_{f}}{m_{0}-m_{f}}$$ (2)

Where K(t) is disintegration coefficient; $\;m_{0}$ is initial balance reading (g); $m_{t}$ is balance reading at a specific immersion time (g); and $m_{f}$ is balance reading before test (g).

#### 2.2.4. Scrub test.

**Fig 3:**
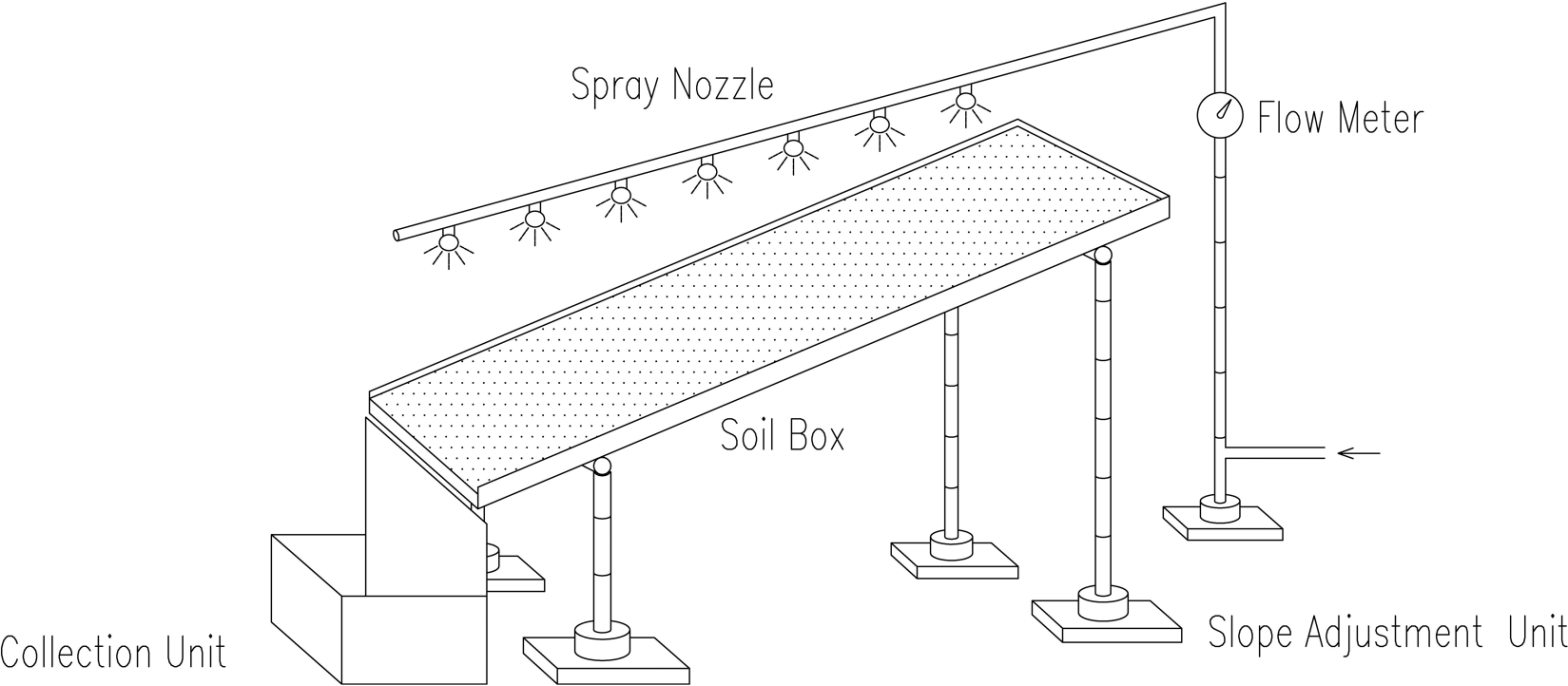
Schematic diagram of slope erosion.

In this experiment, 2500g of sieved soil was weighed and placed into a soil box (As shown in Fig 3) measuring 30 cm (length) × 20 cm (width) × 5 cm (height). The soil was then compacted to a uniform height of 3 cm. To simulate varying conditions, NPCA aqueous solutions with concentrations of 0.5%, 1.0%, 2.0%, and 3.0% were prepared. These were evenly applied to the sandy soil surface at a rate of 2L/m^2^. For control purposes, reference samples were treated with an equivalent volume of water. All samples were subsequently maintained for three days at room temperature to standardize treatment conditions.

The test was designed to replicate the rainstorm conditions typical of the Guangzhou area [32]. A rainfall intensity of 10 mm/min was used, with the soil box positioned at a slope angle of 30°, and the rainfall duration set at 10 minutes. The primary metric for assessing the erosion resistance of the soil was the scour rate, which was calculated using the following formula:


$$W_{a}=\frac{\Delta m}{m_{d}}\times 100\;\%$$ (3)

Where $W_{a}$ is scour rate; Δm is mass of soil lost due to scouring (g); $m_{d}$ is initial dry weight of soil in the box (2500g).

In this formula, a lower scour rate indicates a stronger resistance of the soil to erosion. This calculation allows for a quantitative assessment of the soil's ability to withstand erosive forces under simulated rainstorm conditions.

## 3. Results and disscusions

### 3.1. Reinforcement depth

**Fig 4:**
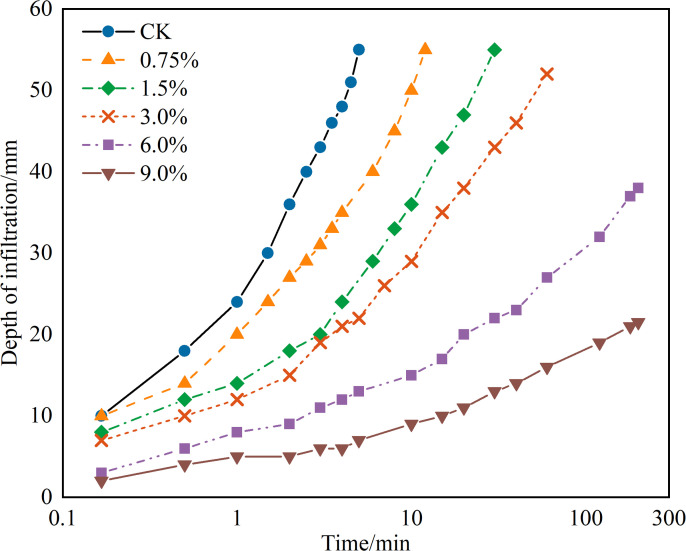
Curve of NPCA aqueous solution infiltration depth over time.

**Fig 5:**
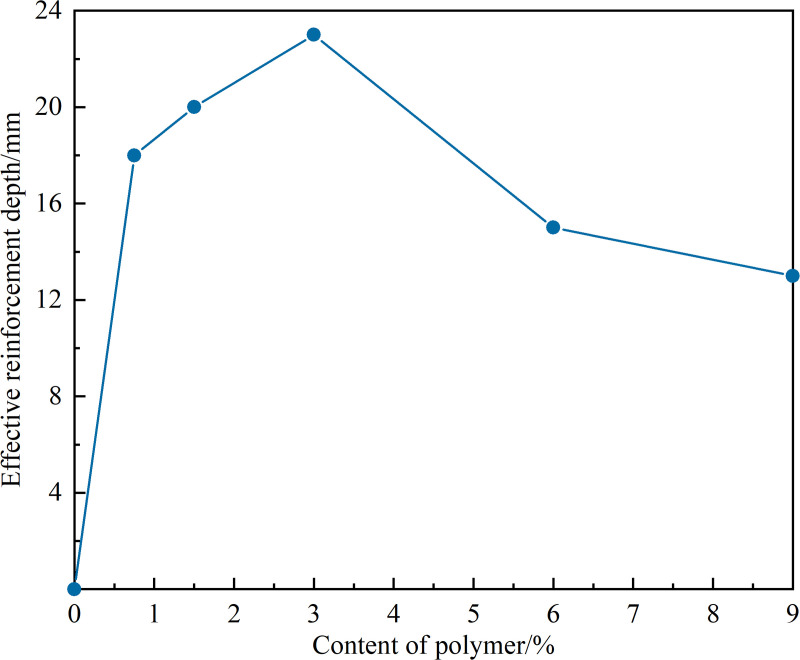
Curve of effective reinforcement depth variation with NPCA dosage.

The diffusion pattern of the NPCA aqueous solution in sandy soil is illustrated in Fig 4, while Fig 5 displays the effective reinforcement depth pattern.

Fig 4 indicates that the NPCA aqueous solution's diffusion rate in sandy soil decelerates with an increase in NPCA dosage. For the reference sample (CK), which utilized water for infiltration, complete permeation was achieved within five minutes. In contrast, with NPCA dosages of 0.75%, 1.5%, and 3.0%, the infiltration times were 12, 30, and 60 minutes, respectively. At higher dosages of 6% and 9%, the NPCA solution failed to permeate the entire sample within 3 hours, reaching infiltration depths of only 37 mm and 21 mm, respectively, which correspond to 67% and 38% of the sample's height.

The sandy soil specimens’ residual length after water immersion represents the effective consolidation depth. This is based on the assumption that the portion of the soil that remains intact after soaking is sufficiently bonded and structurally stable, indicating it has undergone effective consolidation. As shown in Fig 5, the disintegrated portion corresponds to the unconsolidated layer, while the remaining segment reflects the stabilized depth. The reference sample disintegrated within one minute of immersion, leaving no residual length. As the NPCA dosage increased, the effective depth of consolidation initially increased, then decreased. The maximum effective depth of reinforcement, 23 mm, was observed at 3% NPCA dosage, while the minimum effective depth, 13 mm, was seen at 9% NPCA dosage, representing only 56.5% of the maximum depth.

Upon analyzing the NPCA aqueous solution's diffusion and the effective consolidation depth, it is evident that NPCA polymer latex particles disperse in water and permeate deeply into the soil, driven by the dispersing medium. Throughout this diffusion process, the viscous latex particles continuously bond with sand particles and fill pore spaces. The higher the NPCA dosage, the more pronounced the effect of latex particles bonding and filling pores, eventually reaching a limit state where pores are completely filled. This inhibits further diffusion of the particles into deeper soil layers, leading to an accumulation on the surface. At an NPCA dosage of up to 3% (equivalent to an NPCA concentration of 17%), the variation in diffusion depth is minimal. However, increasing the NPCA concentration enhances the bonding effect on deeper sandy soil particles, improving soil particle cohesion.

### 3.2. Permeability of sandy soils

**Fig 6:**
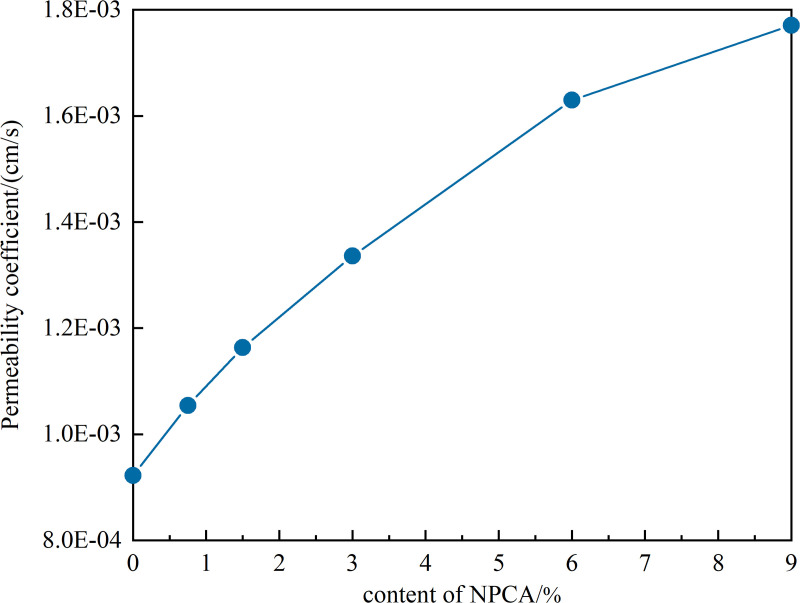
Curve of sandy soil permeability coefficient variation with NPCA dosage.

The impact of NPCA dosage on the permeability of sandy soil is depicted in Fig 6. As the NPCA addition increased, a gradual enhancement in sandy soil permeability was observed. The permeability coefficient for the control sample (CK) was 9.22 × 10^-4 cm/s. For the NPCA-reinforced samples at 0.75%, 1.5%, 3%, 6% and 9% concentrations, the coefficients were 1.05, 1.16, 1.34, 1.63 and 1.77 × 10^-3 cm/s, respectively. These values represent increases by factors of 1.14, 1.26, 1.45, 1.77, and 1.92 compared to the control.

The underlying mechanism involves the formation of a water film on sand particle surfaces in the reference sample, which attracts adjacent particles and generates a pseudo-adhesive force. This process compacts the sand and reduces its volume. With NPCA reinforcement, the inter-particle mesh membrane connections enhance the sand's overall stability. The more NPCA added, the more complete the mesh membrane formation, allowing the sand to retain its original pore structure upon water exposure due to the mesh's supportive role. The permeable nature of the mesh membrane facilitates water passage, thereby enhancing the sandy soil's permeability.

**Fig 7:**
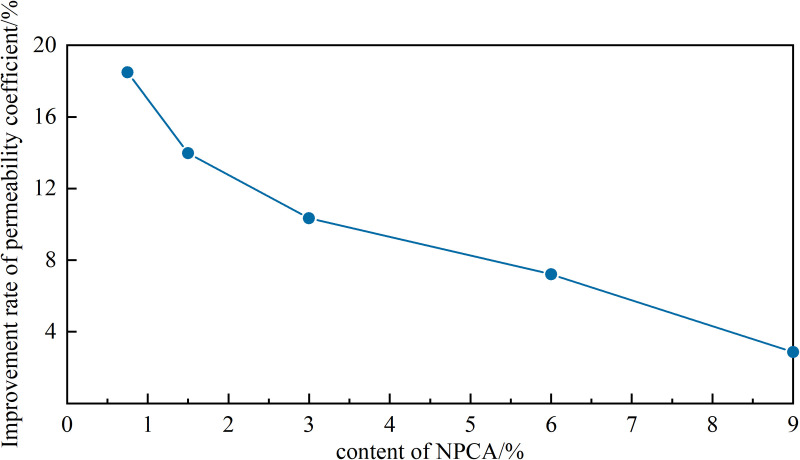
Curve of sandy soil permeability coefficient increase rate variation.

Fig 7 illustrates the rate of permeability improvement in sandy soils with different NPCA additions. Experiments show that NPCA additions over 3% lead to complete pore filling, with excess latex particles obstructing circulation and accumulating on the surface. Consequently, the unit increment contribution of NPCA to sandy soil permeability varies. The rates of permeability coefficient improvement for the 0.75%, 1.5%, 3%, 6% and 9% NPCA-reinforced samples were 18.49%, 17.19%, 15.1%, 12.79% and 10.21%, respectively. Sandy soil permeability is primarily dependent on its stabilized pore condition. Excessive NPCA results in thicker inter-particle mesh membranes, which, while increasing overall stability, do not alter the already stabilized pore structure very much. This finding aligns with the results from the effective reinforcement depth experiments.

### 3.3. Stability of sandy soils in water

**Fig 8:**
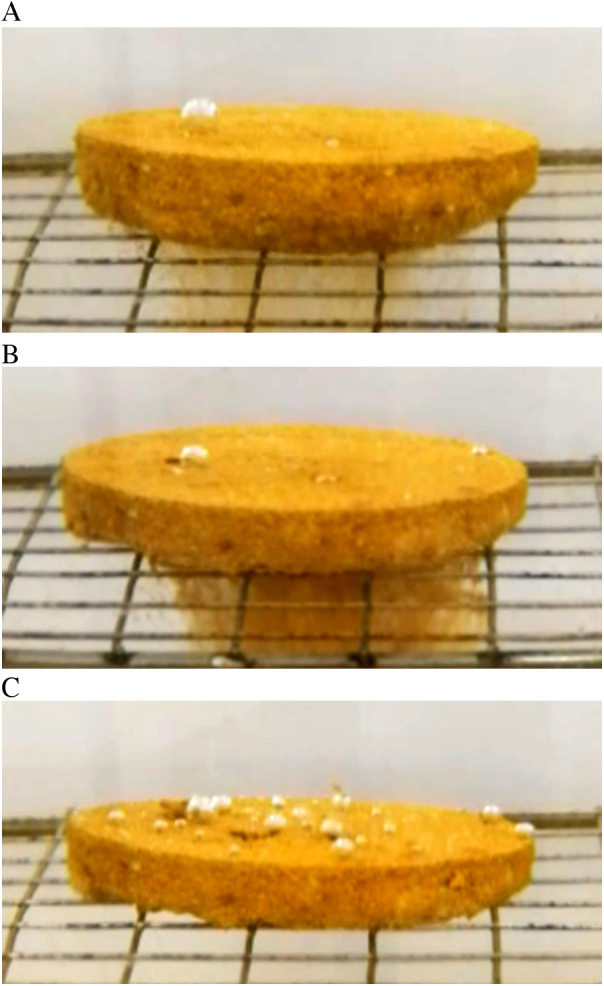
Disintegration process of 3% NPCA reinforced sandy soil specimens. (**a**) Initial state (**b**) Disintegration state (**c**) Final state.

The study investigates the disintegration patterns of sandy soil specimens, specifically those reinforced with NPCA, when immersed in water. This process is depicted in Fig 8, illustrating the initial, disintegration, and final states of 3% NPCA-reinforced sandy soil specimens.

**Fig 9:**
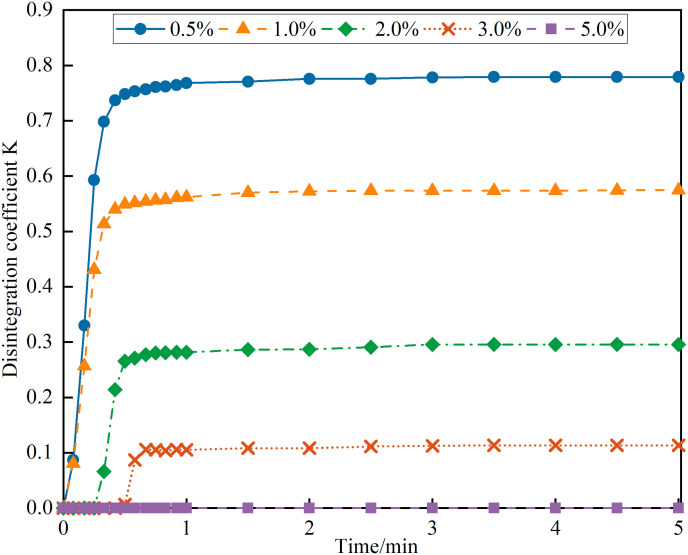
The curve of the disintegration coefficient over time.

Fig 9, exploring the relationship between disintegration coefficient and time, reveals that for sandy soil samples within a maximum reinforcement depth (mass ratio <3%, concentration <17%), there is a notable decrease in disintegration coefficients with increasing NPCA concentrations. Specifically, the disintegration coefficients for samples reinforced at 0.5%, 1.0%, 2.0% and 3.0% concentrations were 0.78, 0.57, 0.29556 and 0.11, respectively. Remarkably, the 3.0% reinforced sample's disintegration coefficient is just 1/7 of the 0.5% reinforced sample, underscoring the effectiveness of NPCA in enhancing soil particle bonding and improving soakage stability.

It is worth noting that the control group (CK), consisting of untreated sandy soil, disintegrated almost immediately upon water immersion. The rapid structural collapse occurred within a few seconds, resulting in an extremely high disintegration coefficient and an ultra-high instantaneous disintegration rate. Due to the absence of sufficient measurable time points, the CK sample could not be effectively plotted in the time-dependent disintegration curve. However, the comparative values of CK have been included in Table X to highlight the contrast in performance. This observation further confirms the critical role of NPCA in significantly enhancing the water stability and cohesion of sandy soils.

**Fig 10:**
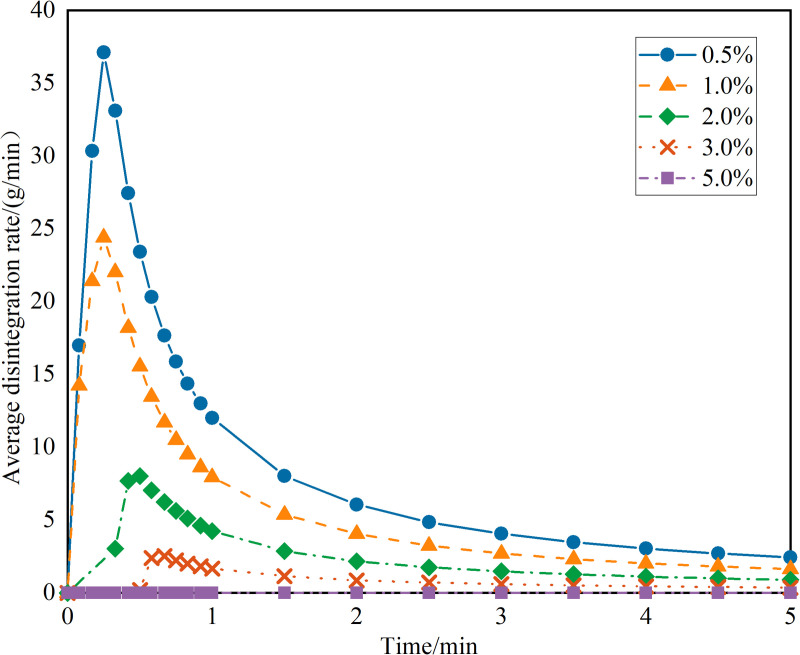
The curve of average disintegration rate over time.

Fig 10 shows the average disintegration rate of sandy soil specimens after water immersion, demonstrating a pattern of an initial increase followed by a decrease. The peak disintegration rate occurs within the first minute of immersion. In samples with increased NPCA concentration (within the maximum reinforcement depth addition, concentration <17%), there's a gradual decrease in the maximum average disintegration rate. Notably, the maximum average disintegration rate for the 0.5% reinforced sample was 37.12 g/min, while the 3.0% reinforced sample decreased dramatically to 2.52 g/min, which is only 6.8% of the 0.5% reinforced sample. Additionally, as NPCA concentration increases, the time to reach peak disintegration rate extends, indicating an enhanced disintegration capacity and prolonged stabilization of sandy soils post-immersion (Table 4).

**Table 4:** Disintegration characteristics at various NPCA dosages.

NPCA(%)	Max Average Disintegration Rate (g/min)	Disintegration Coefficient
0(CK)	≫ 50	≈1
0.5	37.12	0.78
1	24.4	0.57
2	8.02	0.3
3	2.52	0.11
5	≈0	≈0

### 3.4. Erosion resistance of sandy soils

This section presents the results of scour tests conducted on sandy soil slopes, as detailed in Table 5. The table illustrates a significant decrease in the scour rate of these slopes correlating with increased NPCA concentrations.

**Table 5:** Results of sandy soil slope scour tests.

Test Group	Content of ADNB/(g/m^2^)	Weight of dry soil/g	Weight of washout soil/g	Erosion rate Wa/%
CK	0	2500	1224.7	49.0
C-0.5%	10	2500	391.1	15.6
C-1.0%	20	2500	314.9	12.6
C-1.5%	30	2500	223.5	8.9
C-2.0%	40	2500	169.9	6.8

**Fig 11:**
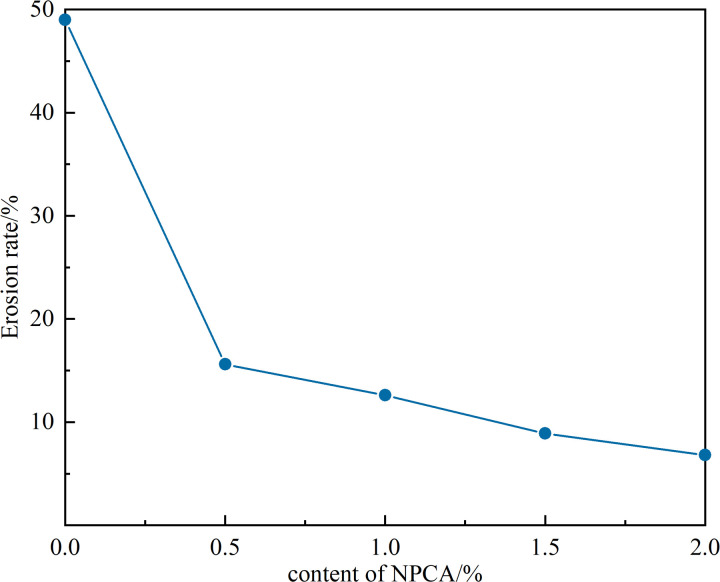
Erosion rate of slope surface.

From Table 5 and Fig 11, it is observed that the scour rate dramatically declines with higher NPCA dosages. The reference sample, denoted as CK, exhibited a high scour rate of 49.0%. In contrast, the sandy soil slope reinforced with 0.5% NPCA (10g/m^2^ dosage) showed a significantly reduced scour rate of 15.6%, which is 68% lower than the CK sample. More notably, the slope reinforced with a 2.0% NPCA concentration (40g/m2 dosage) displayed a further reduced scour rate of only 6.8%.

**Fig 12:**
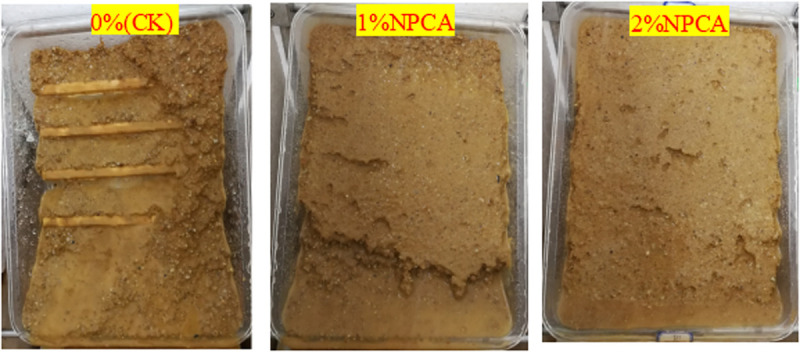
Slope surface erosion patterns under different NPCA treatments.

As shown in Fig 12, a comparative analysis of the scour patterns reveals marked differences. The slope in the control group (CK) exhibited severe spalling, accompanied by visible hollowing beneath the slope surface, indicating weak resistance to runoff-induced erosion. In stark contrast, slopes treated with NPCA aqueous solution developed a protective shell-like layer. This layer effectively mitigated spalling, demonstrating a highly efficient scour-resistant effect. The NPCA treatment thus not only reduces the scour rate but also substantially enhances the structural integrity of the slope under erosive conditions.

### 3.5. Scanning electron microscope analysis

In this section, we explore the results of a Scanning Electron Microscope experiment focusing on the interaction between NPCA and sandy soil. NPCA, primarily composed of hydrolysis-resistant polyester, appears in latex particle form within an aqueous solution. This solution exhibits a notable viscosity.

When applied to sandy soil slopes, NPCA’s aqueous solution effectively influences the soil's physical structure. The latex particles in the solution bond with the soil particles, covering their surface and filling the pore spaces under the water-dispersed liquid's flowing action. As the water evaporates, the polymer chains within the latex particles expand and solidify. This process creates an elastic bonding structure that envelops the soil particles and fills loose pore spaces, thereby improving particle cohesion and enhancing intergranular structural integrity.

**Fig 13:**
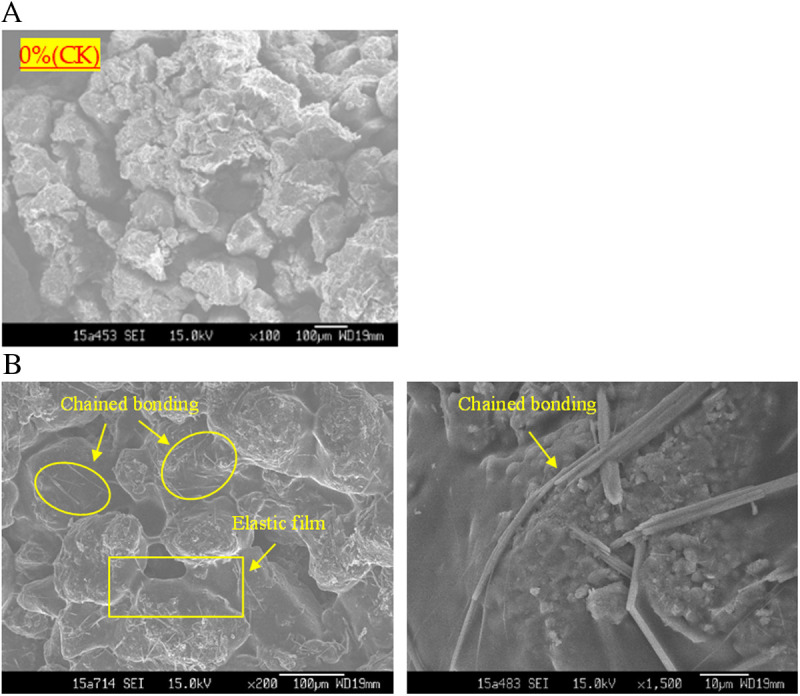
Polymer ‘Reinforcing Chains’ on sandy soil particles.

The influence of this process is significantly affected by the concentration of latex particles. At higher concentrations, a continuous and elastic film is formed both on the surface of sand particles and within the pore spaces. This film exhibits notable toughness and maintains a certain level of permeability. In contrast, at lower concentrations, the latex particles generate stretched, entangled, and interwoven polymer chains, which arrange themselves into a mesh-like network of longitudinal and transverse “reinforcing chains” on the sand particle surfaces (as illustrated in Fig 13). This network behaves as an elastic layer, effectively enhancing the structural integrity of the sandy soil.

### 3.6. Infrared Spectroscopy Experiment

This study further investigates the interaction between NPCA and sandy soil particles through infrared spectroscopy analysis. The focus was on identifying changes in the functional groups, specifically hydroxyl (-OH) and carboxyl (-COOH), present on the polymer chain of NPCA.

**Fig 14:**
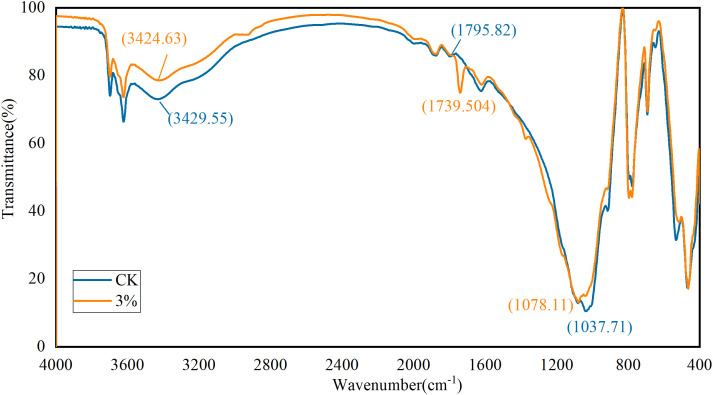
Comparison of infrared spectroscopy spectra of sandy soil.

The analysis compared the infrared spectra of sandy soil before and after modification with NPCA (as shown in Fig 14). Notably, within the range of 3200 cm^-1^ to 3800 cm^-1^, the absorption peaks at 3697 cm^-1^ and 3622 cm^-1^, attributed to the OH stretching vibrations of mineral crystals, remained largely unchanged. This suggests that the mineral structure of the sandy soil was not significantly altered.

However, a shift was observed in the OH stretching vibrations induced by adsorbed water. The peak decreased from 3429 cm^-1^ (as shown in Fig 14) to 3424 cm^-1^, moving to a lower wave number. This shift indicates the formation of hydrogen bonds, suggesting a chemical interaction between the NPCA and the sandy soil.

Furthermore, significant changes were observed in the 1200 cm^-1^ to 2000 cm^-1^ range. Notably, a small peak at 1795 cm^-1^ (as shown in Fig 14) transformed into a sharper peak at 1739 cm^-1^, and its intensity increased. Additionally, the absorption peak at 1622 cm^-1^ decreased to 1620.77 cm^-1^, indicating a shift to a lower wave number. This change is indicative of H-O-H deformation vibrations.

These spectroscopic changes highlight the chemical interactions between NPCA and sandy soil particles, particularly the formation of hydrogen bonds and alterations in water molecule interactions.

## 4. Mechanisms of NPCA reinforcement in sandy soil

Sandy soil primarily consists of quartz, a silica oxide with non-ionic crystal characteristics, and contains minimal amounts of clay minerals. Due to the low surface reactivity of quartz and the absence of significant ion exchange capacity, traditional electrostatic or ionic bonding interactions are negligible. Scanning electron microscopy (SEM, Fig 13) and Fourier-transform infrared spectroscopy (FTIR, Fig 14) were used to investigate the interaction mechanisms between NPCA and sandy soil. The results suggest that NPCA reinforces sandy soil mainly through two mechanisms: direct physical adsorption and mechanical anchoring.

(1) Direct Adsorption Theory
  ① Van der Waals Force

The NPCA emulsion consists of nano-sized latex particles suspended in water, exhibiting high viscosity. During the curing process, as water evaporates, the latex particles move closer to the surface of the non-polar, uncharged quartz particles. This reduced inter-particle distance enhances van der Waals attractions, promoting adhesion at the interface.

SEM images (Fig 13) reveal the formation of a continuous polymer film coating the surface of sand particles after curing. This morphological change is absent in untreated samples, confirming the physical attachment of NPCA to the quartz surfaces.

② Hydrogen Bonding

Although sandy soil lacks clay minerals, trace amounts of mica, a silicate with exposed hydroxyl groups, are present. NPCA contains hydrophilic functional groups, such as –OH and –COOH, which can form hydrogen bonds with mica particles.

FTIR analysis (Fig 14) shows a red shift in the –OH absorption peak (~3300 cm ⁻ ¹) and enhanced intensity in the –COOH region (~1700 cm ⁻ ¹) after mixing with soil, suggesting the formation of hydrogen bonds.

This effect becomes more pronounced with increased NPCA content, indicating a concentration-dependent stabilization effect that improves cohesion among soil particles.

(2)Mechanical Anchor Theory

The second reinforcement mechanism involves the mechanical interlocking of NPCA within the pore structure of sandy soil. Sandy soils are characterized by large intergranular voids and rough particle surfaces, which reduce natural cohesion but provide space for physical entrapment.

**Fig 15:**
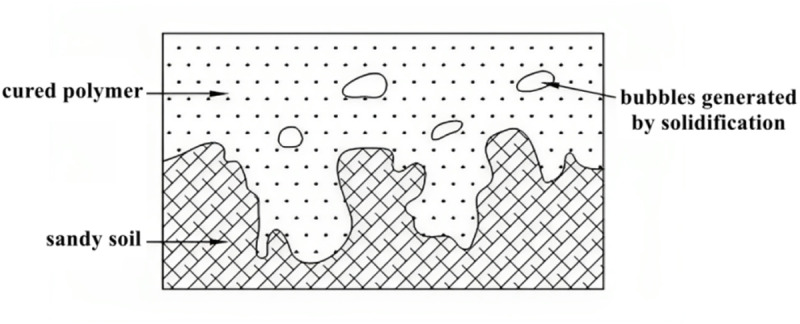
NPCA as mechanical anchors in sandy soil pores.

During mixing, NPCA infiltrates these voids in a dispersed phase. As it cures and transitions from liquid to gel to solid, it forms polymer bridges and ‘nails’ between particles, effectively anchoring the matrix together (Fig 15).

This anchoring effect increases inter-particle friction and resists external deformation, contributing significantly to the mechanical integrity of the soil.

1. Influence of NPCA on sandy soil stability for ecological slope protection
  (1) Effect of NPCA consolidation depth on vegetation cover

Initial vegetation phases, namely emergence and nutrient growth, are critical for slope stabilization. High germination rates, influenced by seed burial depth, determine the eventual vegetation cover. Standard berm vegetation practices involve planting seeds at a depth of 5–10 mm.

**Fig 16:**
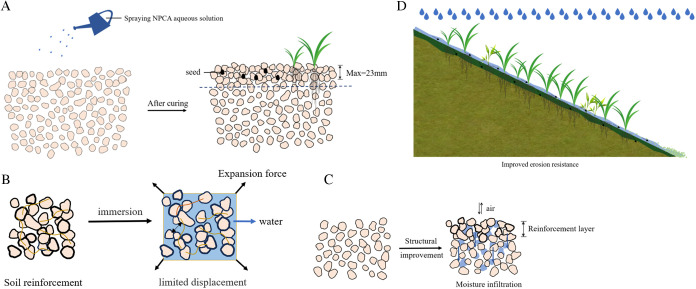
Ecological protection mechanism of NPCA reinforced sandy soil slope.

In this study, the consolidation depth of NPCA was experimentally determined in the first part of the research. The results showed that a 3% NPCA dosage produced a maximum consolidation depth of approximately 23 mm. Importantly, this reinforced layer provides a stable and porous substrate environment that closely aligns with the typical seeding depth. As a result, it effectively supports seed germination and early plant development (as shown in Fig 16(a)).

Moreover, numerous studies [33–35] have demonstrated that improvements in soil structural quality can significantly promote plant establishment and growth. Therefore, these findings provide strong evidence that enhancing soil structure through NPCA application is an effective strategy to support vegetation establishment and ecological recovery.

(2) Effect of NPCA on the stability of sandy soils in water by leaching

NPCA’s molecular bonding and flocculation enhance the water stability of sandy soil. When applied to the soil surface, NPCA forms a resilient elastic membrane and a “reinforcing chain,” creating an additional intergranular connecting force(as shown in Fig 16(b)). This structure restricts particle displacement under external forces, thereby improving mechanical stability. Moreover, the water stability of the elastic membrane and “reinforced chain” ensures the soil's enhanced resistance to water.

(3) Effect of NPCA on the permeability of sandy soils

NPCA structurally improves sandy soil, enhancing permeability and permeability stability. Microscopic experiments show that NPCA’s van der Waals force and hydrogen bonding interlink soil particles into small agglomerates. This linkage rearranges the soil structure from particle-to-particle to agglomerate-to-agglomerate connectivity, increasing pore space and improving soil structure(as shown in Fig 16(c)). Consequently, the polymer elastic membrane and “reinforced chain” stabilize this structure, improving water permeability and reducing surface runoff erosion.

(4) Effect of NPCA on the erosion resistance of sandy soils

The surface film formed by NPCA improves the top layer's resistance to spattering and surface erosion. Uniformly applied NPCA enhances the structure of top soil particles, forming a protective layer of polymer-soil particles. This layer acts as a shield against kinetic energy from raindrops and reduces runoff-induced soil loss. The elastic membrane's toughness and permeability, combined with the protective shell, mitigate surface runoff scouring(as shown in Fig 16(d)).

## 6. Conclusion

(1) NPCA Application and Reinforcement Depth: When the aqueous solution of nano-organic polymer NPCA is sprayed onto sandy soil, the NPCA latex particles disperse into deeper layers, bonding with particles and filling pore spaces. We observed a maximum effective reinforcement depth of 23 mm at a 3% NPCA dosage (17% concentration).
(2) Improvements in Soil Properties with NPCA: Within the range of optimal reinforcement depth (mass ratio <3%, concentration <17%), increasing NPCA dosage enhances the permeability and stability of sandy soils. We noted a decrease in the disintegration coefficient, reduction in the peak average disintegration rate, extension of immersion stabilization time, and improved resistance to scouring.
(3) Molecular Bonding and Aggregate Formation: NPCA bonds to sand particles and fills pore spaces through adhesion. Post-curing, it forms an elastic connection with sand particles via van der Waals forces and hydrogen bonding. The resulting elastic membrane and polymer ‘reinforced chains’ act like anchors in the pore spaces, increasing intergranular connectivity and overall soil stability.
(4) Ecological Protection Benefits of NPCA: NPCA enhances the ecological protection of sandy soil slopes by providing a stable matrix environment. This improvement is achieved through structural enhancements that increase soil permeability, internal bonding and polymer flocculation for better soakage stability, and surface film formation that bolsters resistance to spattering and surface erosion.
